# Occurrence and antimicrobial resistance of pathogenic *Escherichia coli* and *Salmonella* spp. in retail raw table eggs sold for human consumption in Enugu state, Nigeria

**DOI:** 10.14202/vetworld.2016.1312-1319

**Published:** 2016-11-28

**Authors:** O. Josephine Okorie-Kanu, E. Vivienne Ezenduka, C. Onwuchokwe Okorie-Kanu, L. Chinweokwu Ugwu, U. John Nnamani

**Affiliations:** 1 Department of Veterinary Public Health and Preventive Medicine, Faculty of Veterinary Medicine, University of Nigeria, Nsukka, Enugu State, Nigeria; 2 Department of Veterinary Pathology, Michael Okpara University of Agriculture, Umudike, Abia State, Nigeria

**Keywords:** antimicrobial agents, farms, pathogenic *Escherichia coli*, retail outlets, *Salmonella species*, table eggs

## Abstract

**Aim:**

This study was conducted to investigate the occurrence of pathogenic *Escherichia coli* and *Salmonella* species in retail raw table eggs sold for human consumption in Enugu State and to determine the resistance of these pathogens to antimicrobials commonly used in human and veterinary practices in Nigeria.

**Materials and Methods:**

A total of 340 raw table eggs comprising 68 composite samples (5 eggs per composite sample) were collected from five selected farms (13 composite samples from the farms) and 10 retail outlets (55 composite samples from the retail outlets) in the study area over a period of 4-month (March-June, 2014). The eggs were screened for pathogenic *E. coli* and *Salmonella* species following standard procedures within 24 h of sample collection. Isolates obtained were subjected to *in-vitro* antimicrobial susceptibility test with 15 commonly used antimicrobials using the disk diffusion method.

**Results:**

About 37 (54.4%) and 7 (10.3%) of the 68 composite samples were positive for pathogenic *E. coli* and *Salmonella* species, respectively. The shells showed significantly higher (p<0.05) contaminations than the contents for both microorganisms. The eggs from the farms showed higher contamination with pathogenic *E. coli* than eggs from the retail outlets while the reverse was the case for *Salmonella* species even though they were not significant (p>0.05). The organisms obtained showed a multiple drug resistance. They were completely resistant to nitrofurantoin, sulfamethoxazole/trimethoprim, penicillin G and oxacillin. In addition to these, *Salmonella* spp. also showed 100% resistance to tetracycline. The pathogenic *E. coli* isolates obtained were 100% susceptible to gentamicin, neomycin, ciprofloxacin, and amoxicillin-clavulanic acid while *Salmonella* spp. showed 100% susceptibility to erythromycin, neomycin, and rifampicin. Both organisms showed varying degrees of resistance to streptomycin, amoxicillin, vancomycin, and doxycycline.

**Conclusion:**

From the results of the study, it can be concluded that the raw table eggs marketed for human consumption in Enugu State, Nigeria is contaminated with pathogenic *E. coli* and *Salmonella* species that showed multiple drug resistance to antimicrobial agents commonly used in veterinary and human practice.

## Introduction

Poultry is an important farm species in almost all countries including Nigeria. It is an important source of animal protein and can be raised with limited capital. More than 150 million chickens are raised intensively annually, as a source of food for meat, eggs or both [1].

Even though eggs and their products have been found to contain high levels of cholesterol and so attract little patronage by adults, they still remain a very important food for children. Apart from the direct consumption of eggs as food, eggs are also used in the preparation of several commercial and home-made products such as mayonnaise, cake, sandwich, hamburger, and pastries. In terms of nutritive value, poultry ranks 2^nd^ to cow milk [2]. The nutritive value of egg makes it vulnerable to microbial contamination.

Foodborne diseases cause an estimated 48 million illnesses and 3000 deaths in the United States [3]. This could be higher in developing countries like Nigeria where little or no control measures are put in place. Salmonellosis and colibacillosis are the most common and leading cause of foodborne outbreaks worldwide [4]. They constitute a major public health burden and represent a significant cost in medical care in many countries. *Salmonella* spp. and in particular *Salmonella* Enteritidis outbreaks in humans are very often linked to the consumption of contaminated eggs or food containing contaminated eggs [5]. The bacterium infects the eggs by either vertical transmission during the development of the egg within the ovary or horizontal transmission through trans-shell contamination [6]. Nontyphoidal salmonellosis is one of the most common causes of foodborne diarrheal disease worldwide and remains a major public health problem in many parts of the world [7]. It is often implicated in over 60% of cases of human salmonellosis in Europe and North America [8], Latin America, the Middle East, and Africa [9], also in countries such as India [10], Japan [11], and the United States [12]. Several studies had documented isolation of nontyphoidal *Salmonella* from humans and poultry in different parts of Nigeria [13,14]. Outbreaks of salmonellosis caused by *Salmonella* Gallinarum, *Salmonella* Pullorum, *Salmonella* Typhimurium, and *Salmonella* Enteritidis have also been reported [13-15]. *Escherichia coli* is a common inhabitant of the intestinal tract of mammals and it can be easily spread through water, soil, and food. Most strains are harmless [16] but others are capable of causing either intestinal or extra-intestinal diseases [16]. The strain, *E. coli* O157:H7 has been reported to be pathogenic to humans causing bloody diarrhea and hemolytic uremic syndrome [17]. Several materials such as litter, fecal matter, dust in poultry houses, rodent droppings, water, and feeds among others have been implicated as possible sources of *E. coli* in poultry [18].

Antimicrobial resistance has become an increasing public health issue worldwide [19]. Antimicrobial resistance, especially of pathogenic bacteria, has been partly attributed to the misuse of antimicrobial agents in medicine and agriculture [19]. Since their discovery in the 1940s, antibiotics have been widely used in both human and veterinary medical practice [20]. Antibiotics are used by the poultry industry and poultry veterinarians to enhance growth and feed efficiency and to reduce disease. Antibiotic usage has facilitated the efficient production of poultry, allowing the consumer to purchase, at a reasonable cost, high-quality meat and eggs [21]. Furthermore, poultry feeds have been presumed to have a high content of microorganism sequel to the manufacturing and distribution processes to adversely affect the growth and reproduction of poultry. This has, therefore, necessitated the incorporation of antimicrobial agents into poultry feeds which reduce the microbial load in the field and in the gastrointestinal tracts of the poultry, kill or inhibit the growth of infectious organisms or reduces the intensity of antibiotic resistance, thereby improving the gross growth and quality of poultry [22].

The underlying assumption is that poultry feeds are sterile with the incorporation of antimicrobial agents. However, this incorporation poses the emergence or variability of some resistant bacteria either through genetic or nongenetic mechanisms [23]. These drugs (or congeners) are also used in poultry production. The husbandry practice used in the poultry industry and the widespread use of medicated feeds in broiler and layer operations made poultry a major reservoir of antimicrobial resistant *Salmonella* and *E. coli* [24].

The effectiveness of currently available antibiotics is decreasing due to the increasing number of resistant strains causing infections [25]. The reservoir of resistant bacteria in food animals implies a potential risk for transfer of resistant bacteria, or resistant genes from food animals to humans [26]. In developed countries, stringent control of antibiotic use coupled with effective surveillance of antibiotic resistance patterns in the population, have successfully reduced the prevalence of antibiotic resistance to these agents [27]. The situation in the developing countries like Nigeria is however different, where antimicrobial agents are readily available to people in local drug stores without prescription [28]. Such practice has led to misuse of antibiotic resistance among isolates from animal and food sources [13]. Thus, there is a need to constantly monitor susceptibility trends in bacterial agents of economic and public health importance. Since foods of animal origin including meat and eggs are implicated as the most common cause of foodborne infections, a microbial load of table eggs is routinely evaluated before retail selling in developed countries such as USA, Canada, and Japan. However, such practices are not followed in developing countries like Nigeria.

The study, therefore, was designed to investigate the occurrence and antimicrobial resistance of pathogenic *E. coli* and *Salmonella* spp. in retail raw table eggs in Enugu State, Nigeria.

## Materials and Methods

### Ethical approval

The study was approved by the University Committee on Medical and Scientific Research Ethics, University of Nigeria.

### Study area

The study was conducted in Enugu State, which is situated in the Southeastern part of Nigeria [29]. The climate of Enugu State is tropical continental characterized by cool, humid wet seasons and cold or hot dry seasons. Poultry (meat and egg) production is the major business of the rural dwellers. This can be attributed to the high demand for eggs and chicken in Enugu State as a result of the increased number of government parastatals/establishments and the workers. Hence, consumption of poultry meat and eggs is part of the food habit/culture of the population. The state comprises 17 local government areas (LGAs), out of which 5 (Udenu, Nsukka, Igbo-Etiti, Enugu North, and Igbo-Eze North) were selected by simple random sampling technique for the study. The samples were collected from 5 farms and 10 retail outlets including the markets.

### Sampling procedure

Sampling of eggs from farms and retail outlets was carried out from March to June 2014. 65 (5 eggs per composite) freshly laid table eggs which comprise 13 composite samples were collected from 5 poultry farms (1 farm per selected LGA) and 275 (55 composite samples) raw eggs displayed for sale were purchased from 10 retail outlets (2 retail outlets per selected LGA). A total of 340 eggs comprising 68 composite samples were screened for *Salmonella* and pathogenic *E. coli*. The eggs were collected in properly labeled sterile containers and transported within 2 h of collection to Veterinary Public Health Laboratory of the Faculty of Veterinary Medicine, University of Nigeria for analysis within 24 h of collection.

### Processing of egg samples

Sterile gloves were worn to handle egg samples from each source. For egg shells from a composite sample of 5 eggs, one sterile swab, moistened in sterile distilled water, was applied to the surface of each egg. The swabs applied to 5 egg shells were submerged in 5 ml sterile distilled water as “shell wash.”

For egg content (yolk and albumin) samples, a pool of 5 eggs was submerged in 70% ethanol for 5 min. Thereafter, the pointed ends of the eggs were flamed for 5 s with a Bunsen burner for further disinfection. A sterile scalpel blade was used to make a small hole on the shell through which the egg content was aseptically emptied into a sterile container. The contents of each composite sample were mixed thoroughly for 2 min using a vortex mixer. After which the resulting mixture was inoculated into appropriate enrichment broths and media.

### Isolation and identification of bacteria

About 1 ml of “shell wash” of each composite sample and 10 ml of each composite egg contents were inoculated separately into 90 ml of buffered peptone water. They were incubated at 37°C for 18-24 h for pre-enrichment.

### Isolation of *Salmonella* spp.

About 1 ml each of the pre-enriched “shell wash” and the content was inoculated separately into 10 ml each of Rappaport-Vassiliadis medium and incubated at 42°C for 24-48 h for enrichment. A loopful each of the enriched “shell wash” and contents was sub-cultured onto brilliant green agar and incubated at 37°C for 24 h. Presumptive *Salmonella* spp. colonies that appear opaque or pinkish against red background were picked and stocked in nutrient agar (NA) slants at 4°C for further identification by Gram-staining and biochemical tests.

### Isolation of pathogenic *E. coli*

A loopful each of the pre-enriched “shell wash” and content was sub-cultured separately onto sorbitol MacConkey and incubated for 18-24 h at 37°C. Colorless colonies were picked and subcultured onto eosin methylene blue agar. Suspected pathogenic *E. coli* colonies that produced greenish metallic sheen was further confirmed following biochemical tests.

### Biochemical characterization of isolates

Biochemical characterization was based on standard techniques [30]. Suspected *Salmonella* and pathogenic *E. coli* isolates were subjected to urease, triple sugar iron (TSI), and simmons citrate biochemical tests after Gram-staining. Molecular identification of the *Salmonella* serovars and pathogenic *E. coli* as well as their virulence factors/pathotypes could not be done due to lack of funds and facilities. Following the biochemical characterization, confirmed *Salmonella* and pathogenic *E. coli* isolates were kept at 4°C in NA slants for determination of antimicrobial susceptibility tests.

### Evaluation of the *in-vitro* susceptibility of the isolates to antimicrobial agents

All the isolates identified as *Salmonella* spp. and pathogenic *E. coli* were tested for their susceptibility to 15 antimicrobial agents with the following disk contents: Neomycin (N) - 30 µg, doxycycline (D) - 30 µg, rifampicin (RD) - 25 µg, sufamethoxazole/trimethoprim (SXT) - 25 µg, amoxicillin (AML) - 10 µg, vancomycin (VA) - 30 µg, nitrofurantoin (F) – 300 µg, penicillin-G (P) – 10 µg, ciprofloxacin (CIP) – 30 µg, erythromycin (E) – 15 µg, gentamicin (CN) – 30 µg, streptomycin (S) – 20 µg, tetracycline (TE) – 30 µg, oxacillin (OX) – 5 µg, and amoxicillin-clavulanic acid (AMC) (30 µg) using the disk diffusion method described by Bauer *et al*. [31] and based on the recommendations of Clinical Laboratory Standard Institute (CLSI) [32].

Briefly, *Salmonella* spp. and *E. coli* isolates were recovered from NA slants by culturing isolates on appropriate culture media. Two to three colonies of the isolates were inoculated separately into 5 ml tryptone soya broth (Oxoid, UK) and incubated for 24 h at 37°C. Mueller-Hinton agar (MHA) plates were prepared according to manufacturer’s instructions. Sterile swab sticks were used to collect the broth cultures and inoculated evenly on the MHA plates. The plates were kept to dry at room temperature for 10 min. The antimicrobial discs were carefully picked with sterile forceps and carefully placed on the plates. Minor pressure was applied on the discs to ensure proper contact with the medium.

The plates were incubated at 37°C for 18 h. Susceptibilities to the antimicrobial discs were observed as a clear zone of inhibition around the discs. The inhibition zone diameters were measured to the nearest millimeter and interpreted following chart provided by CLSI [32].

### Data analysis

The association between the occurrence of the organisms (*Salmonella* spp. and pathogenic *E. coli*), and the source was analyzed with Chi-square test of independence and Fisher’s exact test using SPSS package version 16. Significance was accepted at p≤0.05.

## Results

### Occurrence of *Salmonella* spp. and pathogenic E. coli

Out of the 68 composite samples of eggs obtained from farms and retail outlets in the study area, 37 (54.4%) were positive for pathogenic *E. coli* while 7 (10.3%) yielded *Salmonella* spp. Out of the 37 (54.4%) that were positive for pathogenic *E. coli*, 26 (38.2%) were from the shell while 11 (16.2%) were from the content. Out of the 7 (10.3%) *Salmonella* positive samples, 6 (8.8%) were from the shell while 1 (1.5%) was from the content (Table-1).

**Table 1:** Occurrence of *Salmonella* spp. and pathogenic *E. coli*.

Source (Organism)	Number of composite sample	Shell (%)	Content (%)	Total (%)
Retail outlets	55			
*E. coli*		18 (32.7)	10 (18.2)	28 (50.9)
*Salmonella*		6 (8.8)	1 (1.5)	7 (12.7)
Farms	13			
*E. coli*		8 (61.5)	1 (7.7)	9 (69.2)
*Salmonella*		0 (0)	0 (0)	- (0)
Total	68			
*E. coli*		26 (38.2)	11 (16.2)	37 (54.4)
*Salmonella*		6 (8.8)	1 (1.5)	7 (10.3)

*E. coli*=*Escherichia coli*

From the retail outlets alone, 55 composite samples (275 eggs) were collected. Out of which 18 (32.7%) were positive for pathogenic *E. coli* from shell while 10 (18.2%) were positive for *E. coli* from content, making a total of 28 (50.9%) pathogenic *E. coli* samples. All the *Salmonella* isolates obtained were from the retail outlets (Table-1).

From the farms, 13 composite samples (65 eggs) were collected. Out of which 8 (61.5%) yielded pathogenic *E. coli* from the shell while 1 (7.7%) yielded pathogenic *E. coli* from the content.

### Biochemical tests

37 isolates of pathogenic *E. coli* were positive to TSI test and negative to both simmons-citrate and urease tests while 7 *Salmonella* spp. isolates were positive to both TSI and simmons-citrate tests but negative to urease test.

### *In-vitro* susceptibilities of the isolates to 15 antimicrobial agents

The percentage susceptibilities of pathogenic *E. coli* and *Salmonella* isolates to the 15 antimicrobials tested were observed. All the pathogenic *E. coli* isolates were susceptible to 4 antimicrobial agents, namely, gentamicin, neomycin, ciprofloxacin, and AMC acid and were resistant to nitrofurantoin, sulfamethoxazole-trimethoprim, penicillin G, and oxacillin. 89% of the pathogenic *E. coli* isolates were resistant to rifampicin, 84% to streptomycin, erythromycin and tetracycline, 81% to amoxicillin, 78% to vancomycin and 57% to doxycycline. 10 resistance patterns were recorded. All the isolates exhibited a multiple drug resistance. The resistance patterns observed is shown in Table-2.

**Table 2:** Antimicrobial resistance patterns of the pathogenic *E. coli* isolated.

F- SXT- P- OX -RD- S- E- TE- AML- VA- DO	19 isolates
F- SXT- P- OX- RD- S- E- TE- AML- VA	7 isolates
F- SXT- P- OX- RD- S- AML- VA	3 isolates
F- SXT- P- OX- RD-TE-AML- DO	1 isolate
F- SXT- P- OX- RD- E- TE- AML	1 isolate
F- SXT- P- OX- S- E- TE- AML	2 isolates
F- SXT- P- OX- RD- TE- DO	1 isolate
F- SXT- P- OX- RD- E- AML	1 isolate
F- SXT- P- OX- RD- S- AML	1 isolate
F- SXT- P- OX- E	1 isolate

*E. coli*=*Escherichia coli*, DO=Doxycycline, RD=Rifampicin, SXT=Sufamethoxazole/trimethoprim, AML=Amoxicillin, VA=Vancomycin, F=Nitrofurantoin, P=Penicillin-G, E=Erythromycin, S=Streptomycin, TE=Tetracycline, OX=Oxacillin

All the *Salmonella* spp. isolates were susceptible to three antimicrobial agents, namely, erythromycin, neomycin and rifampicin, and where, just like *E. coli*, resistant to nitrofurantoin, SXT, penicillin G and oxacillin in addition to tetracycline. 85% of the *Salmonella* spp. isolates were resistant to streptomycin, 71% to amoxicillin and AMC acid, 57% to gentamicin, 42% to vancomycin, and doxycycline and 29% to ciprofloxacin. Four resistance patterns were observed, and all the isolates exhibited a multiple drug resistance. The resistance patterns observed is shown in Table-3.

**Table 3:** Antimicrobial resistance patterns of the *Salmonella* species isolated.

F- SXT- P- OX- TE- S- AML- AMC- CN- VA- DO	3 isolates
F- SXT- P- OX- TE- S- AML- AMC	2 isolates
F- SXT- P- OX- TE- S- CN	1 isolate
F- SXT- P- OX- TE-	1 isolate

DO=Doxycycline, RD=Rifampicin, SXT=Sufamethoxazole/trimethoprim, AML=Amoxicillin, VA=Vancomycin, F=Nitrofurantoin, P=Penicillin-G, E=Erythromycin, CN=Gentamicin, S=Streptomycin, TE=Tetracycline, OX=Oxacillin

## Discussion

Microbial contamination of egg has an important outcome on the poultry industry especially with regard to international trade. It also has a serious public health implication worldwide with regard to the transmission of illness to humans which could result in mild symptoms or life-threatening conditions.

The study showed high contamination of eggs marketed for human consumption in Enugu State, Nigeria with *Salmonella* and pathogenic *E. coli*. These microorganisms were detected from both the shell and the content. This agrees with USDA [33] which stated that microorganisms can be found both on the outside and inside of egg. This may be due to the fact that the egg and the feces share the same environment within and outside the laying bird. Feces known to be highly contaminated with microbes could contaminate the egg as it passes through the cloaca to be laid. On the other hand, the presence of microorganisms inside the egg could be as a result of the presence of the pathogens within the hen’s ovary or oviduct before the formation of the shell around the yolk and albumin. These pathogens could have originated from the feed [34] of the birds or from the environment. Fecal contaminants on the shell of freshly laid eggs could also gain access into the eggs through the pores of the egg. Ansah *et al*. [35] reported that as eggs stay longer outside after lay, the natural barrier against microorganisms on the shell breaks down, thus reducing the eggs ability to resist the penetration of microorganisms into the content through the shell.

In this study, it was observed that eggs from retail outlets recorded significantly higher contamination (p<0.05) with *Salmonella* spp. than eggs obtained from farms. This could be attributed to the unhygienic conditions in the markets and retail outlets where these eggs are openly displayed for sale, the improper/unhygienic handling of eggs by farm workers, retailers and buyers, the prolonged poor storage conditions of the eggs during sale and high salt content of feed fed to birds which could result in watery feces and fecal pasting/soiling of the eggs. Humphrey and Whitehead [36] reported that bacterial growth in the eggs stored at 18-30°C could be explained by the decreased integrity of the vitelline membrane, which accelerates leakage of the yolk contents, and in turn causes a rapid bacterial growth in the albumin after 6-10 days.

This result however disagrees with the result of Adesiyun *et al*. [37] who reported significantly higher contamination of the eggs from farms with *Salmonella* spp. than retail outlets. The reason for this could be due to the additional washings of eggs carried out at the sale outlets in the study area to make the eggs appear clean and acceptable to the consumers which are not done in Nigeria.

On the other hand, it was observed that eggs from farms recorded higher contamination with pathogenic *E. coli* than eggs obtained from retail outlets, even though it was not statistically significant (p>0.05), it is of significant public health importance. In general, *E. coli* is a normal flora of the gastrointestinal tract of animals and birds. Therefore, when eggs are freshly laid in the farms, there is high contamination of the eggs with *E. coli*, but as the eggs move from the farms to the retail outlets, these eggs are touched indiscriminately by the buyers, thereby inadvertently carrying some of the *E. coli* on the eggs and resulting in the reduction in the quantity of *E. coli* on the eggs at retail outlets as observed in this study.

Furthermore, the growth rate of *E. coli* has been reported to decrease at room temperature [38]. When eggs are freshly laid as seen in farms, they are at optimum temperature for the growth of pathogenic *E. coli*. However, when the eggs are moved to the retail outlets, there is decreased growth of the pathogenic *E. coli* due to decrease in temperature below the optimum temperature for growth, hence the observed significant increase in contamination of eggs at farms than at retail outlets with *E. coli*.

The study also recorded significantly higher contamination (p<0.05) from the shell than from the content of the egg. The isolation rates of the pathogenic *E. coli* and *Salmonella* from the shell were 38.2% and 8.8%, respectively, while from the content, the isolation rates were 16.2% and 1.5%, respectively. The high isolation rate of pathogenic *E. coli* from the shell could be attributed to the fact that *E. coli* are generally normal intestinal flora of humans and birds, and so could easily contaminate egg shell through feces and subsequently the content. However, strains such as enterohemorrhagic *E. coli* (O157:H7) are pathogenic for humans [39]. Moreso, coliforms (*E. coli*) populations can be used as a measure of food quality and sanitary processing conditions. The presence of this bacterium in large numbers on the eggs isolated from both retail outlets and farms indicates the poor sanitary conditions of these places [40].

The *Salmonella* spp. got from this study was detected on the eggs from retail outlets. This shows that the contamination could be from an internal and external sources as almost all the eggs sampled were from deep litter system in addition to the other factors such as unhygienic conditions in the markets and retail outlets where these eggs are openly displayed for sale, the prolonged poor storage conditions of the eggs during sale and the unhygienic handling of eggs by farm workers, retailers, and buyers.

Of particular public health importance is the presence of these microorganisms (pathogenic *E. coli* and *Salmonella* spp.) in the egg contents also meant for consumption by immunocompromised persons. However, the isolation rate of *Salmonella* spp. in this study (8.8% from shell and 1.5% from egg content) is lower than the 13.5% reported by Salihu *et al*. [41] in Sokoto State while the isolation rate of pathogenic *E. coli* from the shell (38.2%) is however higher than that obtained by Mai *et al*. [42] in retail outlets in Jos, Plateau State where the researcher reported an isolation rate of 27.5%.

The threat of antimicrobial resistance is growing at an alarming pace, perhaps more rapidly in developing countries [43,44]. Aside from the abuse of antimicrobials, a number of circumstances converge to this rapid growth and spread, ranging from the biological traits that bacteria deploy to face antimicrobials to regulatory and financial issues behind antimicrobial abuse. Bacterial resistance to antimicrobial drugs is one of the most serious jeopardies to global public health.

Multiple drug resistance was observed in all isolates of pathogenic *E. coli* and *Salmonella* spp. tested in this study. Both micro-organisms showed 100% resistance to nitrofurantoin (F), sulfamethoxazole-trimethoprim (SXT), penicillin G (P) and oxacillin (OX). *Salmonella* in addition showed 100% resistance to tetracycline. This can be attributed to the indiscriminate use of antimicrobials in clinical and veterinary practices in Nigeria. It has been reported that inappropriate use of antimicrobials in livestock may result in the development of resistance among bacteria in these animals and their products [45]. Reports from different parts of Nigeria have observed temporal trends in the prevalence of resistance among enteric organisms [46]. Resistance to commonly used antimicrobials, including SXT, ampicillin, tetracycline, and chloramphenicol has shown increasing prevalence in the last 25 years [46]. In Nigeria, poultry industry is at infancy and small/local farmers and ill persons easily purchase drugs over the counter without prescription by a veterinarian or a medical practitioner. This encourages the misuse and abuse of these antimicrobials, especially the cheap/affordable ones [47]. Another reason for the resistance could be because these antimicrobials have been in use in the poultry industry over a long period of time, and so microorganisms could have developed resistance to them over time. Andersen *et al*. [48] reported that resistance to penicillins and other β-lactam drugs by *Salmonella* and other enteric pathogens is attributable to the acquired ability of the strains to produce β-lactamase, while resistance to tetracycline is highly associated with the acquisition and expression of efflux pumps that reduce toxic levels of the drug in the bacterial cells.

The multidrug resistance observed in this study could also be mediated by genetic mobile elements such as plasmids, transposons, and integrons as seen in the case of oxacillin resistance. Coincidentally, *Staphylococcus aureus* isolated during the course of this study showed a high degree of resistance to oxacillin. Thus could have transferred resistance to the enteric organisms through the genetic mobile elements or vice versa.

High level of antimicrobial resistance was similarly reported in SXT and tetracycline by Ekundayo and Ezeoke [49] among *Salmonella* isolates obtained from eggs got from farms in Abia State while Tafida *et al*. [50] similarly reported high resistance to penicillin G and oxacillin by *Salmonella* from retail beef in Zaria, Kaduna State, Nigeria.

Generally, nitrofuran drugs commonly employed for the treatment of salmonellosis and other bacterial infections in poultry are banned for use in livestock feed in many countries, including Nigeria, because of their mutagenic potentials [51]. Surprisingly, both micro-organisms showed 100% resistance to nitrofurantoin. This shows that in addition to the uncontrolled use of antimicrobials in poultry production by farmers, nitrofurans, even though banned, may still be in use by commercial feed millers in the production of commercial livestock feeds marketed in the study area. It is therefore necessary to conduct a surveillance study to determine the occurrence of antimicrobials (especially the banned ones) in livestock feed in Nigeria.

The generally high prevalence of resistance to other antimicrobials (tetracycline, rifampicin, streptomycin, erythromycin, amoxicillin, vancomycin and doxycycline) can also be attributed to the uncontrolled/widespread use of these antimicrobials as mainly growth promoters since the farmers have unlimited access to these agents and their use [52]. This however disagrees with the reports of Tafida *et al*. [50] who reported susceptibility to SXT, tetracycline, and nitrofurantoin, and Ekundayo and Ezeoke [49] who reported susceptibility of nitrofurantoin. This may be due to the drug use pattern in their study areas which may be different from that in present study area.

Furthermore, *E. coli* obtained in this study were observed to be 100% susceptible to gentamicin, neomycin, ciprofloxacin and AMC acid while *Salmonella* showed 100% susceptibility to erythromycin, neomycin, and rifampicin. These may be attributed to the high cost of these drugs which results in their reduced demand/use in poultry production. Farmers are mindful of the cost of production and so purchase the readily affordable drugs to maximize profit thereby unintentionally causing the development of resistance among the cheap and affordable drugs by their misuse and abuse behavior.

## Conclusion

Based on the results of the study, it can be concluded that retail raw table eggs marketed for human consumption in Enugu State, Nigeria is contaminated with foodborne pathogens *E. coli* and *Salmonella*. Also, the isolated pathogens were 100% resistance to nitrofurantoin, sulfamethoxazole/trimethoprim, penicillin G and oxacillin which are commonly used antimicrobial agents in veterinary and human practices. This could have a significant public health consequence if these microorganisms are transmitted to humans through food chain.

## Authors’ Contributions

OJO, EVE, and COO got the concept of the work, designed the study, did the data analysis and interpretation. LCU, EVE, UJN and OJO performed the lab analysis of the samples and drafted the articles. All authors read, corrected and revised the manuscript.
